# S100B Affects Gut Microbiota Biodiversity

**DOI:** 10.3390/ijms24032248

**Published:** 2023-01-23

**Authors:** Vincenzo Romano Spica, Federica Valeriani, Massimiliano Orsini, Maria Elisabetta Clementi, Luisa Seguella, Gianluca Gianfranceschi, Rosa Di Liddo, Gabriele Di Sante, Francesca Ubaldi, Francesco Ria, Giuseppe Esposito, Fabrizio Michetti

**Affiliations:** 1 Laboratory of Epidemiology and Biotechnologies, Department of Movement, Human and Health Sciences, University of Rome “Foro Italico”, 00135 Rome, Italy; 2 Laboratory of Molecular Ecology, Istituto Zooprofilattico Sperimentale delle Venezie, 35020 Legnaro, Italy; 3 Istituto di Scienze e Tecnologie Chimiche “Giulio Natta” SCITEC—CNR, 00168 Rome, Italy; 4 Department of Physiology and Pharmacology—“Vittorio Erspamer”, Sapienza University of Rome, 00185 Rome, Italy; 5 Department of Pharmaceutical and Pharmacological Sciences, University of Padova, 35131 Padova, Italy; 6 Department of Medicine and Surgery, Section of Human, Clinical and Forensic Anatomy, University of Perugia, 06132 Perugia, Italy; 7 Department of Translational Medicine and Surgery, Section of General Pathology, Catholic University of the Sacred Heart, 00168 Rome, Italy; 8 Department of Neuroscience, Catholic University of the Sacred Heart, 00168 Rome, Italy; 9 IRCCS San Raffaele Scientific Institute, Università Vita-Salute San Raffaele, 20132 Milan, Italy; 10 Department of Medicine, LUM University, 70010 Casamassima, Italy

**Keywords:** microbiome, 16S amplicon sequencing, NGS, S100B, mfDNA, Pentamidine, IBD, eubiosis, milk, gut–brain axis, protein-binding domain

## Abstract

This in vivo study in mice addresses the relationship between the biodiversity of the microbiota and the levels of S100B, a protein present in enteroglial cells, but also in foods such as milk. A positive significant correlation was observed between S100B levels and Shannon values, which was reduced after treatment with Pentamidine, an inhibitor of S100B function, indicating that the correlation was influenced by the modulation of S100B activity. Using the bootstrap average method based on the distribution of the S100B concentration, three groups were identified, exhibiting a significant difference between the microbial profiles. Operational taxonomic units, when analyzed by SIMPER analysis, showed that genera regarded to be eubiotic were mainly concentrated in the intermediate group, while genera potentially harboring pathobionts often appeared to be more concentrated in groups where the S100B amounts were very low or high. Finally, in a pilot experiment, S100B was administered orally, and the microbial profiles appeared to be modified accordingly. These data may open novel perspectives involving the possibility of S100B-mediated regulation in the intestinal microbiota.

## 1. Introduction

S100B is a calcium-binding protein, which, in the central nervous system (CNS), is concentrated in astrocytes, although it is also expressed in other neural and extra-neural cell types [1,2]. It has also been shown to be expressed in enteroglial cells, which essentially play the equivalent role of astrocytes in the enteric nervous system (ENS) [3,4]. When secreted, S100B has been shown to be trophic at nanomolar concentrations, as well as toxic at micromolar levels, behaving as a damage/danger-associated molecular pattern (DAMP) protein under neuroinflammatory conditions [1,2,5]. Thus, the enteric S100B may be regarded as a diffusible cytokine participating in immune–inflammatory processes in the gut and/or a trophic factor for the district [3,6,7,8].

Although S100B has been intensively studied in the CNS, it is present also in different tissues and biological fluids, including blood, urine, and saliva [2,9,10]. In the gut, it is reasonably secreted by enteroglial cells, but it may also be taken in with food, having been shown to be present in milk, possibly as a trophic factor, supposed to play a role in newborn development [11,12]. The protein has also been shown to be present in human feces, being reduced in inflammatory bowel disease (IBD) patients [13,14]. In light of the consideration that phenomena occurring at the gut surface play a role in the critical interface between the body and the environment, the S100B protein, being present in the bowel as an intrinsic molecule and as a constituent of food and in feces, reasonably may participate in the physiological and pathological processes of the gut mucosa [15]. In this light, it might also be regarded as a putative actor in the microbiota/gut communication machinery. With the purpose of exploring this possibility, we performed an in silico study [16], showing the differential capability of S100B to interact with the proteome of a healthy and IBD microbiota, suggesting a possible role at the mucosa–microbiota barrier, in the ENS, and in the gut microbiota axis. In continuity with this previous in silico investigation, the present in vivo study in mice addresses the relationship between S100B levels and the microbiota biodiversity, further approaching the hypothesis of a possible interaction at a local level, involving mechanisms related to the gut microflora equilibrium.

## 2. Results

### 2.1. Gut Microbiota Biodiversity Increases with S100B Levels

**Figure 1:**
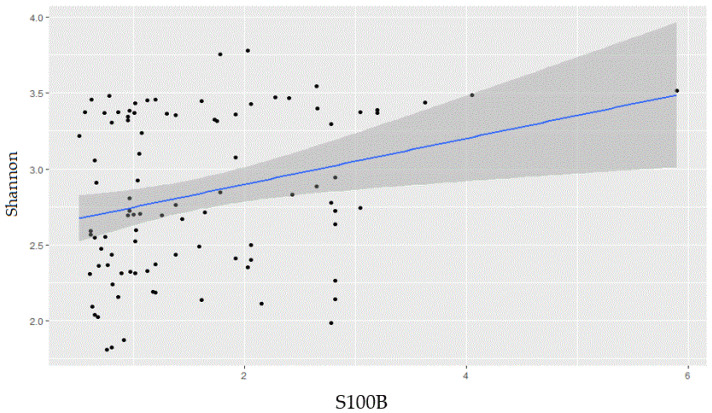
Relationship between concentration of S100B and Shannon alpha-biodiversity index. The scatterplot shows a positive correlation between S100B concentration (ng/mL) and Shannon values. The regression equation was Y = 0.15X + 2.59 without the 0 values (intercept 2.4–2.8).

A total of 66 animals were used in this study to evaluate the relationship between gut microbiota biodiversity and S100B levels. A positive and significant correlation was observed between S100B levels and Shannon values, with a whole correlation coefficient of 2.59 ± 0.4 (R^2^ = 0.97, Figure 1). These findings support the presence of a possible association between the amount of S100B in the gut and the degree of biodiversity observed in the gut microbiota.

### 2.2. S100B Effect on Microbiota Is Reduced by Pentamidine

**Figure 2:**
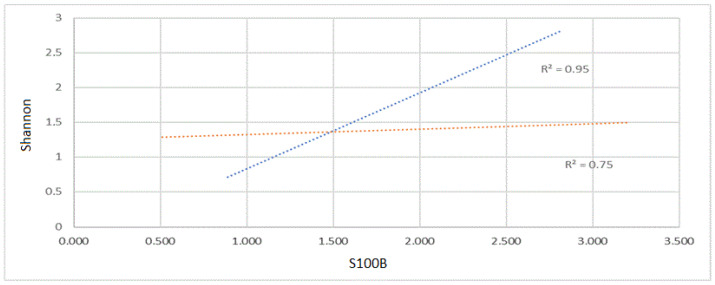
Relationship between S100B concentration and Shannon index in mice treated (*n* = 25) with PTM (red line) and without (n = 28) PTM treatment (blue line). The diagram shows a positive correlation between S100B concentration (ng/mL) and Shannon values without treatment (R^2^ = 0.95) that is reduced after PTM treatment (R^2^ = 0.75).

In order to verify whether the correlation between S100B and biodiversity was influenced by the activity of the protein, the same data (Shannon index and S100B levels) were collected after a treatment with Pentamidine (PTM), a selective inhibitor of S100B function, known to block its protein-binding domain. Interestingly, in those mice that were treated with PTM, the correlation was significantly reduced (R^2^ = 0.75; *p* < 0.05 Figure 2). This observation supports a specific role for S100B active domains in the microbiota biodiversity.

### 2.3. S100B Levels and Microbial Diversity Can Cluster into Three Groups

**Figure 3:**
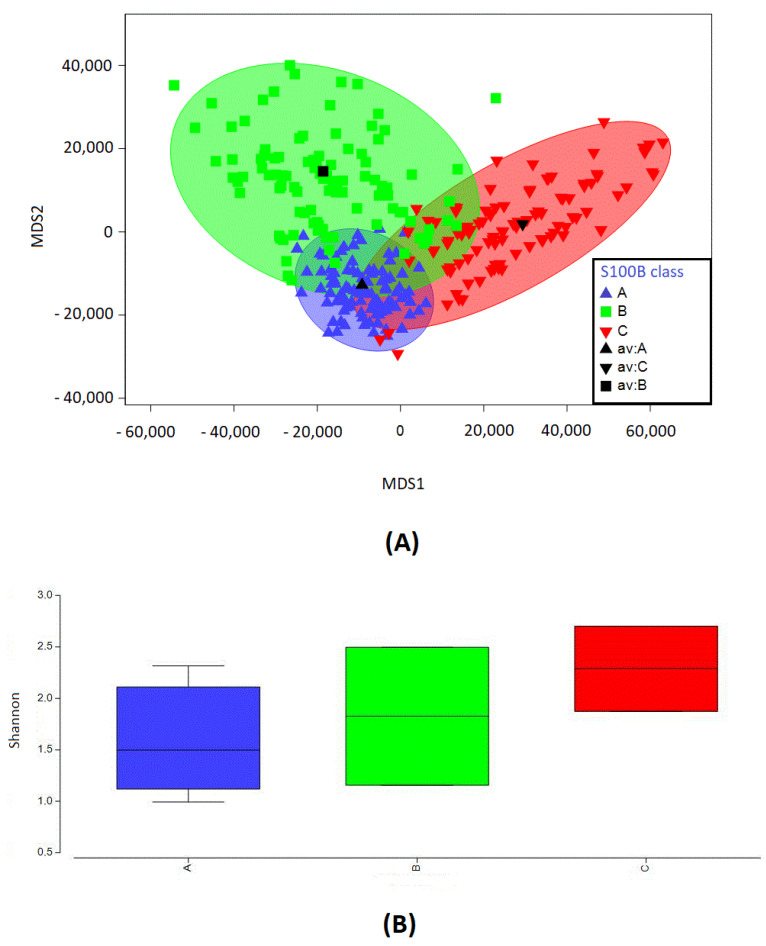
(**A**) Correlation between S100B levels and the microbiota biodiversity clusters samples into 3 groups. Bootstrap average analysis of the microbiota composition in relationship with levels of S100B (A = lower than 1.75 ng/mL, B = between 1.76 and 2.4 ng/mL, C = higher than 2.5 ng/mL). (**B**) Shannon index values with respect to S100B clusters. Box plots represent the distribution of calculated Shannon index for microbiota samples based on the different levels of S100B (A = lower than 1.75 ng/mL, B = between 1.76 and 2.4 ng/mL, C = higher than 2.5 ng mL).

To acquire indications on the stratification of biodiversity based on S100B concentration levels, a statistical analysis was performed on the observed sample via the bootstrap average method (cutoff > 1%, stringency > 97%). Based on the distribution of the S100B protein concentration (ng/mL) in the whole colon tissue (mucosa and lumen content), the microbial profile data clustered into three groups, low (A < 1.75 ng/mL), medium (B = 1.76–2.4 ng/mL), and high (C > 2.5 ng/mL) concentrations, as shown in Figure 3A. Although different minor regions for overlapping arose, three independent groups were clearly identified by the S100B levels. This finding suggests the existence of putative threshold levels for S100B amounts differently influencing the microbiota biodiversity in the gut. Interestingly, a statistically significant difference in the mean value of Shannon was observed between groups A and C (*p* < 0.01), further confirming the linearity between S100B and the Shannon index and the correlation between biodiversity and defined S100B thresholds, as summarized in Figure 3B. Based on multivariate analysis using the bootstrap average, a significant difference (*p* < 0.05) in the microbial profiles of samples from the three groups was confirmed. Microbial profiles of samples included in A and C levels of S100B protein (respectively, S100B values lower than 1.75 ng/mL and higher than 2.5 ng/mL) are dissimilar for 92.5%; meanwhile, A (S100B values lower than 1.75 ng/mL) and B (S100B values between 1.76 and 2.4 ng/mL) are dissimilar for 89%, hinting at some possible physiological threshold levels for the S100B concentration in the gut and their correlation with the microbiota structure.

### 2.4. S100B Clustering and OTU

**Figure 4:**
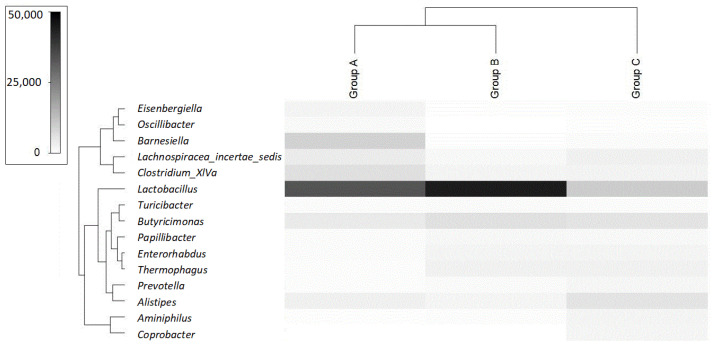
Hierarchical clustering dendrogram on 16S amplicon sequencing data. Dendrogram shows hierarchical clustering of samples based on genus-level classifications. The bar chart under each sample summarizes the relative abundance of its genus-level classifications, as measured in number of reads. Samples are divided based on the different levels of S100B (A = lower than 1.75 ng/mL, B = between 1.76 and 2.4 ng/mL, C = higher than 2.5 ng/mL).

Operational taxonomic units (OTUs) were analyzed by SIMPER analysis, to identify possible differences in the distribution of different microbial species within the three S100B clusters identified by the bootstrap method. The hierarchical clustering of samples was performed based on genus-level classification. Main differences are reported in Figure 4, showing which OTUs significantly contributed to the variations in between-sample diversity in relation to the S100B threshold levels. The significant (*p* < 0.0002) dissimilarities between the groups were 87.64%, 88.76% and 85.51%, respectively, for A versus C, A versus B and C versus B. A consistent difference in the OTU distribution of microbial species was observed in the three clustering groups. Fifteen significant OTUs were identified as being mostly affected by the protein level. A linear positive or negative trend was not observed between the S100B concentration and each specific OTU. However, several OTUs showed higher representation in some of the clustering groups, suggesting a tendency based on the different A, B, C groups of protein concentrations. Some OTUs showed increased representation at the lowest or highest S100B concentrations (group A and C, respectively). Other OTUs, instead, were more represented at an intermediate concentration (group B). OTUs belonging to the phyla of Firmicutes and of Bacteroidetes were both affected by protein levels. For instance, when focusing on Firmicutes at the genus level, a correlation (R^2^ = 0.270, *p* = 0.010) was observed between the presence of *Lactobacillus* in group B (Mean: 45,856 ± 245 reads) versus group A (Mean: 33,342 ± 458 reads) or group C (Mean: 15,312 ± 469 reads). Similarly, *Barnesiella*, belonging to Bacteroidetes, showed an inverted trend (R^2^ = −0.450, *p* < 0.001), with an average value of 686 ± 10 reads versus 18,811 ± 100 and 1584 ± 12 reads, respectively, in group B vs. A and C. Interestingly, a negative correlation was observed between the S100B levels in group A and B (<2.5 ng/mL) and *Clostridium* spp. (R^2^ = −0.358, p < 0.001; A = average value of 6085 ± 338 reads and B = average value of 3500 ± 155 reads) or the Lachospiraceae family (R^2^ = −0.300, *p* < 0.001; A = average value of 3704 ± 174 reads and B = average value of 780 ± 45 reads). The negative association was obtained also for genera of other phyla, such as *Alistipes* spp. (R^2^ = −0.300, *p* < 0.001; A = average value of 3409 ± 194 reads and B = average value of 890 ± 34 reads) within the Bacteroidetes phylum. Meanwhile, a positive association was observed for S100B levels over 2.5 ng/mL for *Butyricimonas* spp. (R^2^ = 0.290, *p* = 0.005) for the Bacteroidetes phylum, with an average value of 3600 ± 205 reads versus 6500 ± 100 and 6100 ± 122 reads, respectively, in group A vs. B and C.

The analysis of the three clusters, previously identified, essentially showed that phyla regarded to be trophic for the gut, and probably the entire organism, such as *Lactobacillus*, were especially concentrated in group B, which contained an intermediate concentration of S100B, while phyla including potentially noxious genera or species, such as *Clostridium*, *Alistipes* or *Barnesiella*, were especially concentrated in group A (average value 6085 ± 338 reads, 3409 ± 194 reads and 18,811 ± 100, respectively), where the concentration of S100B was very low, or group C, where S100B was at a high concentration (average value 4530 ± 153 reads, 6150 ± 100 reads and 1584 ± 12 reads, respectively). The entirety of the observed findings further support a relationship between S100B and the microbiota.

### 2.5. S100B Oral Administration Affects Microbiota

In order to further test the relationship between S100B levels and its influence on the microbiota composition, we orally administered S100B to a restricted number of animals (*n* = 3). The administration of a solution containing S100B (200 µg) for two weeks modified the microbial profiles essentially in accordance with the results shown in Figure 1. The S100B mean level was observed to rise from 0.95 ng/mL to 1.94 ng/mL after oral administration, and Shannon index values tended to increase. Although the sample size was limited, the treated animals showed a consistent trend and a dissimilarity with respect to the controls, with an increase in the gut levels of the protein and several differences in biodiversity (OTU average dissimilarity = 30%) in the fecal samples.

## 3. Discussion

The results show a relationship connecting the enteric levels of S100B, an enteroglial protein that may also be taken in with food [1,2], with the biodiversity of the gut microbiota, which is known to affect a series of parameters, also influencing behavior and the onset of diseases not directly involving the gut [15,16,17,18,19,20,21]. The findings confirm, on an experimental basis, a previous in silico study indicating that proteins participating in the microbiota composition in healthy and IBD subjects putatively interact with S100B domains [16,22,23,24,25]. Interestingly, the relationship between the enteric levels of S100B and the biodiversity of the gut microbiota appears to be lost after the administration of PTM, which is regarded to play an inhibitory role in S100B activity, thus supporting the possibility that S100B activity affects gut microbiota biodiversity (Figure 2). Although, at present, the molecular mechanisms are not fully unraveled, PTM is supposed to bind S100B and interfere with its binding proteins [7], suggesting a possible action on putative ligands present in the gut, both at the mucosal level and within the proteome of the microbiota. Data have been reported indicating that PTM displays a synergy with antibiotics typically restricted to Gram-positive bacteria, yielding effective drug combinations with activity against a wide range of Gram-negative pathogens in vitro, and against systemic *Acinetobacter baumannii* infections in mice [26]. However, no statistical differences were shown after treatment with PTM in both the Gram-positive/-negative ratio and in Firmicutes/Bacteroidetes in the present study. Although many hypotheses might be formulated concerning this effect on PTM, reliable information at present leads us to reasonably attribute it to its inhibitory interaction with S100B [7].

The analysis of the distribution of the microbiota biodiversity based on S100B levels allows the definition of three major clusters, where an intermediate group (group B in Figure 3) includes a larger part of the samples with physiological patterns in the microbiota structure. This observation fits the consideration of a possible trophic role for S100B amounts in the order of nanograms. Interestingly, clusters outside of these ranges (group A or group C) show significant differences and the more prevalent presence of microbial genera including potentially noxious species, such as *Clostridium*, *Alistipes* or *Barnesiella* [27,28,29]. In this respect, species considered to be eubiotic or trophic for the gut [28], and probably for the entire organism, such as *Lactobacillus*, are especially concentrated in intermediate group B, as summarized in Figure 4. Taken together, these observations, even if they do not allow the detailed definition of the phenomenon, reasonably support a relationship between the S100B concentration and the microbiota biodiversity. Therefore, a pilot experiment was added to further evaluate this indication and to observe the effect of S100B oral administration on microbiota biodiversity. Although this was a preliminary test on a limited number of animals, the observed microbiota biodiversity trend was in agreement with the guiding hypothesis of a role of S100B in the intestinal lumen. The rationale for oral S100B administration is based on data indicating that this protein is present in breast milk from human and different mammalian species [12,30,31,32], being also detected in feces [13,16]. Additional experiments will be required to verify these preliminary data.

The impact of S100B on the microbiota, and therefore on other tissues and organs through the gut–brain axis, may open different perspectives for a hypothetical novel role of this protein also in the enteric lumen, interacting with the local microflora and the mucous membrane [33]. The gut–brain axis, indeed, is a complex bi-directional communication route involving the connection between the enteric nervous system (ENS) and the central nervous system (CNS), whose correct functioning is essential to the body’s homeostasis [34,35,36]. In this context, the role of the intestinal microbiota diversity has been identified as a pleiotropic regulator of the intrinsic production of neurotrophins and growth factors that play an autocrine role in protecting the intestinal mucosa and, in a paracrine way, at a CNS level, the regulation of neuronal plasticity in different pathophysiological contexts [37,38,39,40]. In this sense, the microbiota is progressively regarded to be a key regulator even of neurological functions, since it may directly influence the level of brain deriving growth factor (BDNF) in mice [33] and of glial cell line-derived neurotrophic factor (GDNF) in specific brain areas [41]; moreover, on the other hand, its depletion is at the basis of many neurological or psychiatric disorders [42]. Moreover, while the impact of the microbiota on the production of neurotrophins in the intestine and CNS is known, the role of neurotrophins produced by the gut and, specifically, by the ENS at the microbiota level, although believed to be likely probable, is still largely unknown.

The presence of S100B in this district, and its interaction with the microbiota, may be related to its location in enteroglial cells [4], which may secrete the protein as astrocytes in the central nervous system, and/or to its intake with food, where at least its presence in breast milk is demonstrated in human and different mammalian species [12,31,43]. In this respect, it may be relevant to consider how different nutrients can profoundly vary the diversity of the microbiota [28,35]. One of the main foods able to play a role of primary importance in the diversification of the microbiota is breast milk [44], and it is believed that this element has a relevant impact during early childhood up to adult life in the regulation of the bacterial species that populate the intestine ecosystem during life [42,45]. Although S100B production at the gut level has been shown to be markedly upregulated during intestinal inflammation [16], at physiological nanomolar levels, it is one of the main neurotrophins that are present in breast milk, reasonably acting as a signaling molecule for the correct development in the early phases of life [42,46]. However, despite this evidence, we still do not know whether the physiological levels of S100B might be responsible for microbiota-mediated development.

The data of the present work lead us to consider an amplification of the role of the S100B protein in the gut–brain axis, offering a rationale for the presence of S100B among the constituents of natural foods such as breast milk, and opening novel perspectives involving the possibility of S100B-mediated regulation in the intestinal microbiota. This novel perspective might reasonably introduce novel pharmacological therapies that may impact the microbiota diversity by S100B modulation. Further studies will be necessary to evaluate the direct role of S100B in the microbiota.

## 4. Materials and Methods

### 4.1. Experimental Animals and Treatments

A total of 132 fecal samples were collected from female mice (n = 66) of the C57Bl/6 strain (Charles River, Calco, LC, Italy) and included in the experimental set reported in Figure 1. All procedures for animal housing and maintenance were performed in compliance with previous studies [46,47,48]. The age of the animals at the start of the study was between 8 and 12 weeks. The mice were monitored daily, and fecal samples were collected every 15 days for the S100B assay and 16S amplicon sequencing analysis. In order to test the effect of Pentamidine (PTM), a S100B inhibitor, as indicated in Figure 2, PTM was administered intraperitoneally (4 mg/kg) to 25 mice (50 fecal samples) vs. 28 untreated controls (as a part of the previous experimental set involving a total of 66 animals), following a previously established protocol [47]. Additionally, in a pilot test, 300 µL of a solution (phosphate-buffered saline—PBS) containing 200 µg of S100B (Sigma, St. Louis, MO, USA) or of vehicle (phosphate-buffered saline—PBS) was orally administered, respectively, to 3 and 2 C57Bl/6 mice. After 15 days, fecal samples (n = 10) were collected and analyzed for microbiota biodiversity.

### 4.2. S100B ELISA Assay

S100B was quantified in the whole colon tissue (mucosa and lumen content) by using a SimpleStep ELISA^®^ (enzyme-linked immunosorbent assay) kit (Abcam, Cambridge, UK). The kit was used according to the manufacturer’s instructions.

### 4.3. DNA Extraction, 16S Ribosomal DNA (rDNA) Sequencing and Bioinformatics Analysis

Fecal samples (approximately 20–40 mg) were collected and stored at −20 °C, to be processed with a previously validated protocol for DNA extraction from feces [49]. Samples were weighed prior to extraction, and DNA was purified and normalized. The libraries for next-generation sequencing (NGS) were prepared according to the 16S Metagenomic Sequencing Library Preparation Guide (part# 15044223 rev A; Illumina, San Diego, CA, USA). The PCR amplicons were obtained using primers already reported in previous publications [49,50,51]. Tagged PCR products were generated using primer pairs with unique barcodes through a two-step PCR. In this strategy, target primers containing overhang adapters were used in the first PCR reaction to amplify the target gene, and the product was then used in the second PCR using primers containing barcodes. Reactions were carried out on a Techne^®^ TC-PLUS thermocycler (VWR International, LLC, Radnor, PA, USA). Following amplification, 5 μL of PCR product from each reaction was used for agarose gel (1%) electrophoresis to confirm amplification. The final concentration of the cleaned DNA amplicon was determined using the Qubit PicoGreen dsDNA BR assay kit (Invitrogen, Grand Island, NY, USA) and validated on a Bioanalyzer DNA 1000 chip (Agilent, Santa Clara, CA, USA). Then, the library was sequenced on an Illumina ISeq100 platform and 150 bp paired-end reads were generated. A total of 13,465,228 sequence reads were produced after NGS analysis. The number of sequences for each sample ranged from 35,000 to 231,800, leading to the identification of 497 ± 198 OTUs defined at 97% identity. Rarefaction curves were calculated for each sample, showing adequate and reliable sampling and sequencing effort for describing the bacterial community. Analysis of the 16S rDNA gene sequences was performed with Quantitative Insights into Microbial Ecology version 2 (QIIME2, version 2021.4). The resulting ASV sequences were assigned to the Greengenes database using the q2-feature-classifier plugin [52].

### 4.4. Statistical Analysis and Bioinformatics

A total of 130 metagenomic data were analyzed using the R environment for statistical computing (Version 4.0.1) and Primer7 (Primer-e, Auckland, New Zealand). Sequencing data from two samples were at a low level of quality and were not included in the analysis. A differential analysis of the intergroup alpha diversity index was conducted using the R language. Box plots were generated based on the alpha diversity indices, using the ggplot2 package of the R language. Continuous variables were the S100B concentration (ng/mL) and Shannon values. Relationships between continuous variables were compared by Pearson’s correlation. Pearson’s χ^2^ test was used to compare the frequencies of the categorical variables, and non-parametric Wilcox or Kruskal tests were used to compare the two groups under the non-normality assumption. Linear models (ANOVA and linear regression) were performed to evaluate the effects of the variables observed on the concentration of S100B. The S100B protein values in detectable amounts (≥0.1 ng/mL) were analyzed by bootstrap average analysis [53,54]. The groups’ clustering was determined based on their degree of Euclidian distance, considering all species with representation over 1% and using default settings with high stringency conditions (>97%). All analyses were considered statistically significant at a *p* value lower than 0.05, if not differently indicated. To identify OTUs that contributed to differences in between-sample diversity, we performed similarity percentage analyses (SIMPER; Primer-e, Auckland, New Zealand) and determined the significance using Kruskal–Wallis tests (kruskal.test, stats package) with false discovery rate correction using the Benjamini–Hochberg method [55]. Significant differences, at the genus level, were identified by the SIMPER test [56]. We used standard linear methods and adjusted all analyses for treatment and sampling day. OTUs were deemed significant at an adjusted *p* < 0.05, and a trend at 0.10 < adjusted *p* < 0.05. SIMPER results were visualized in heatmaps using plot_heatmap (phyloseq, Primer-e, Auckland, New Zealand).

## Data Availability

The raw sequencing data have been submitted to the NCBI Sequence Read Archive (http://www.ncbi.nlm.nih.gov/sra/ accessed on 1 January 2023) with the project accession number of PRJNA904312.
